# Inflammation affects dynamic functional network connectivity pattern changes via plasma NFL in cognitive impairment patients

**DOI:** 10.1111/cns.14391

**Published:** 2023-08-07

**Authors:** Weina Yao, Huijuan Zhou, Xiao Zhang, Haifeng Chen, Feng Bai, Alzheimer's Disease Neuroimaging Initiative

**Affiliations:** ^1^ Department of Neurology Zhongnan Hospital of Wuhan University Wuhan China; ^2^ Geriatric Medicine Center Taikang Xianlin Drum Tower Hospital Clinical College of Wuhan University Nanjing China; ^3^ Department of Neurology, Nanjing Drum Tower Hospital Clinical College of Traditional Chinese and Western Medicine Nanjing University of Chinese Medicine Nanjing China; ^4^ Geriatric Medicine Center Taikang Xianlin Drum Tower Hospital, Affiliated Hospital of Medical School, Nanjing University Nanjing China; ^5^ Department of Neurology Nanjing Drum Tower Hospital, Affiliated Hospital of Medical School, Nanjing University Nanjing China

**Keywords:** Alzheimer's disease, dynamic functional network connectivity, plasma neurofilament light chain

## Abstract

**Background:**

Plasma neurofilament light chain (NFL) is a biomarker of inflammation and neurodegenerative diseases such as Alzheimer's disease (AD). However, the underlying neural mechanisms by which NFL affects cognitive function remain unclear. In this study, we investigated the effects of inflammation on cognitive integrity in patients with cognitive impairment through the functional interaction of plasma NFL with large‐scale brain networks.

**Methods:**

This study included 29 cognitively normal, 55 LowNFL patients, and 55 HighNFL patients. Group independent component analysis (ICA) was applied to the resting‐state fMRI data, and 40 independent components (IC) were extracted for the whole brain. Next, the dynamic functional network connectivity (dFNC) of each subject was estimated using the sliding‐window method and k‐means clustering, and five dynamic functional states were identified. Finally, we applied mediation analysis to investigate the relationship between plasma NFL and dFNC indicators and cognitive scales.

**Results:**

The present study explored the dynamics of whole‐brain FNC in controls and LowNFL and HighNFL patients and highlighted the temporal properties of dFNC states in relation to psychological scales. A potential mechanism for the association between dFNC indicators and NFL levels in cognitively impaired patients.

**Conclusions:**

Our findings suggested the decreased ability of information processing and communication in the HighNFL group, which helps us to understand their abnormal cognitive functions clinically. Characteristic changes in the inflammation‐coupled dynamic brain network may provide alternative biomarkers for the assessment of disease severity in cognitive impairment patients.

## Graphical abstract

This study investigated the effects of inflammation on cognitive integrity in patients with cognitive impairment through the functional interaction of plasma neurofilament light chain (NFL) with large‐scale brain networks. Our findings suggested the high NFL which may contribute to our clinical understanding of their abnormalities in emotional and cognitive functions.

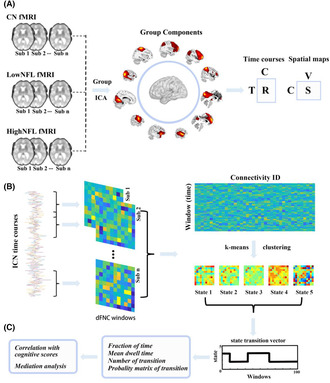

## 1 INTRODUCTION

Alzheimer's disease (AD) is a prevalent neurodegenerative disease characterized by cognitive impairment and dementia in the elderly population, affecting almost 50 million people worldwide.^1^ It has high morbidity and mortality rates and has become increasingly burdensome to families and society in recent years.^2^, ^3^ Significant effort in the past few years has been devoted to the search for biomarkers of AD. Neuropsychiatric disease severity and cognitive impairment are often described and predicted using functional connectivity.^4^ Specifically, plasma NFL is a promising low‐invasive method of assessing various neurologic conditions,^5^, ^6^, ^7^ and plasma NFL has been shown to be a sensitive biomarker of inflammation and chronic neurodegenerative disorders.^8^, ^9^

Measures of functional connectivity describe the functional architecture of the human brain by quantifying the temporal dependence between voxels, brain regions, or network signals.^10^, ^11^ Resting‐state functional magnetic resonance imaging (rs‐fMRI), as a noninvasive measure of brain integrity, can assess brain activity changes and monitor endogenous changes in the blood–oxygen level‐dependent (BOLD) signal under resting conditions. Previous research has confirmed that patients with dementia of AD exhibit functional connectivity abnormalities.^12^, ^13^ Briefly, patients with cognitive disorders exhibited disrupted functional connectivity in several key functional systems compared to healthy elderly individuals.^14^, ^15^ These critical regions include the default mode network (DMN), dorsal attention network (DAN), control network (CON), salience network (SN), and sensorimotor network (SMN). Abnormal functioning of the auditory network (AN) leads to a disruption of memory information, which results in its incorrect processing of internal memory, and these apparently contradictory findings suggest that the presence, severity and stage of illness matter.^16^ However, it has been considered that functional connectivity is static over the entire time period of typical rs‐fMRI protocols but not dynamic. In fact, the human brain is not immutable but obviously a dynamically interactive system. Dynamic functional network connectivity (dFNC) analysis based on resting fMRI shows different connectivity states of the brain over time by summarizing recurring large‐scale connectivity patterns, as well as the mutual transitions between connectivity states.^17^, ^18^, ^19^ Recently, several studies have focused on the dFNC of AD and indicated that the progressively changing connectivity pattern of the dFNC is important for tracking the progression of cognitive impairment and can be considered a biomarker of dementia. In more detail, abnormal connectivity patterns of the whole‐brain dFNC were identified in the early stages of AD, and decreased connectivity among the SMN, visual network (VN), and AN was observed relative to normal healthy individuals.^20^ Another study demonstrated significant associations between dFNC features and cognitive performance on neuropsychological indicators. Importantly, these associations could not be observed between static FNC traits and cognitive scores.^21^

The neurofilament light (NFL) chain is a neural axon cytoskeletal protein that releases NFL into the extracellular space, including peripheral blood, when the pathological process of neural axon injury occurs. In neurodegeneration, NFL can be measured using the ultrasensitive single‐molecule array (SiMoATM) method in the blood due to injury in inflammation‐related acute neuroaxonal injury.^6^ Those who experience longer treatment interruptions are more likely to experience central nervous system inflammation and neuronal damage.^22^ It is a promising blood marker for neurodegenerative diseases.^23^, ^24^ Moreover, plasma biomarkers are the easiest to use in clinical applications, where PET imaging or cerebrospinal fluid lumbar puncture is not yet popular due to low accessibility and open examination. The levels of plasma NFL are related to the preclinical stages of AD, and previous studies have indicated that the level of NFL correlates with brain structure in patients with cognitive impairment.^25^ Briefly, plasma NFL in patients with cognitive impairment has been linked to neuroimaging measures such as hippocampal volume and cortex thickness, as well as cognitive function.^26^ The longitudinal association between plasma NFL and white matter atrophy progressively involved periventricular regions throughout cognitively impaired subjects and appeared to propagate from the temporal lobe.^27^

However, whether and how the changes in plasma NFL could modulate the association between dFNC and cognitive function remain unclear. In the present study, we investigated the moderating effect of plasma NFL on the relationship between dFNC (i.e., dFNC state and dFNC graph theory) and neuropsychological scales in patients with cognitive impairment. First, we hypothesized that plasma NFL could modulate the association between dFNC and cognition, specifically, at the microscopic level, since patients with cognitive impairment have poorer cognitive control than healthy controls. Therefore, these patients may rely on the temporal variability of brain networks that respond to cognitive function to control cognitive‐related activities. Second, considering the possible effects of plasma NFL on cognitive scales, we hypothesized that changes in dynamic functional connectivity could directly affect patients' cognitive dysfunction or indirectly cause cognitive impairment mediated through plasma NFL.

## 2 METHODS AND MATERIALS

### 2.1 ADNI database

Data used in the preparation of this article were obtained from the Alzheimer's Disease Neuroimaging Initiative (ADNI) database (http://adni.loni.usc.edu). The ADNI is a multicenter, longitudinal neuroimaging study launched in 2003 as a public–private partnership led by Principal Investigator Michael W. Weiner, MD. The primary goal of the ADNI has been to identify whether serial MRI, PET, other biological markers, and clinical and neuropsychological assessments would study the pathogenesis and prevention of AD. Since 2003, the ADNI has been further followed by the ADNI‐GO and ADNI‐2 and has recruited over 1500 older adults (aged 55–99) over three phases (ADNI‐1, ADNI‐GO, and ADNI‐2) from over 50 sites in the United States and Canada. ADNI participants consist of cognitively normal older individuals, people with early or late mild cognitive impairment (MCI), and people with AD. The study was approved by the institutional review boards of all participating centers, and written informed consent was obtained from each participant or authorized representative. A detailed description of the full inclusion and exclusion criteria for the ADNI is provided in the Appendix S1.

### 2.2 Participants

In this study, all subjects were obtained from the ADNI database, including 139 participants. The participants included 29 cognitively normal (CN), 55 low plasma neurofilament light chain (LowNFL), and 55 high plasma neurofilament light chain (HighNFL) patients from whom the resting‐state fMRI data were downloaded from the ADNI. In addition, the present study matched the groups by age, education level and sex, and LowNFL and HighNFL, included patients with different stages of cognitive impairment, such as early MCI (EMCI), late MCI (LMCI), and AD. For grouping, plasma NFL values above and below the median were classed as HighNFL and LowNFL, respectively.

### 2.3 Clinical and neuropsychological measurement

Demographic characteristics and neuropsychological assessment data were downloaded from the ADNI database (http://adni.loni.usc.edu). For the primary analyses, all subjects were subjected to a thorough physical and cognitive examination by ADNI or BLSA study personnel. ADNI subjects were evaluated using the Mini‐Mental State Examination (MMSE), Montreal Cognitive Assessment (MoCA), Clinical Dementia Rating (CDR), Alzheimer's Disease Assessment Scale‐Cognitive Section (ADAS), and Functional Activities Questionnaire (FAQ) as general cognition and the Ray Auditory Verbal Learning Test (RAVLT) as a marker of episodic memory. Full information regarding the ADNI inclusion and exclusion criteria can be accessed at http://adni.loni.usc.edu/.

### 2.4 Plasma NFL

During the course of the study, plasma NFL was available for all patients included. Plasma NFL concentrations were measured using an NFL kit (NF light; UmanDiagnostics) and then transferred to an ultrasensitive single‐molecule array platform using a homemade kit (Simoa Homebrew Assay Development Kit; Quanterix Corporation). A 6.7 ng/L was the lower limit of quantification, and 1620.0 ng/L was the upper limit. All measurements fell within the limits of quantification.

### 2.5 MRI scanning

**FIGURE 1:**
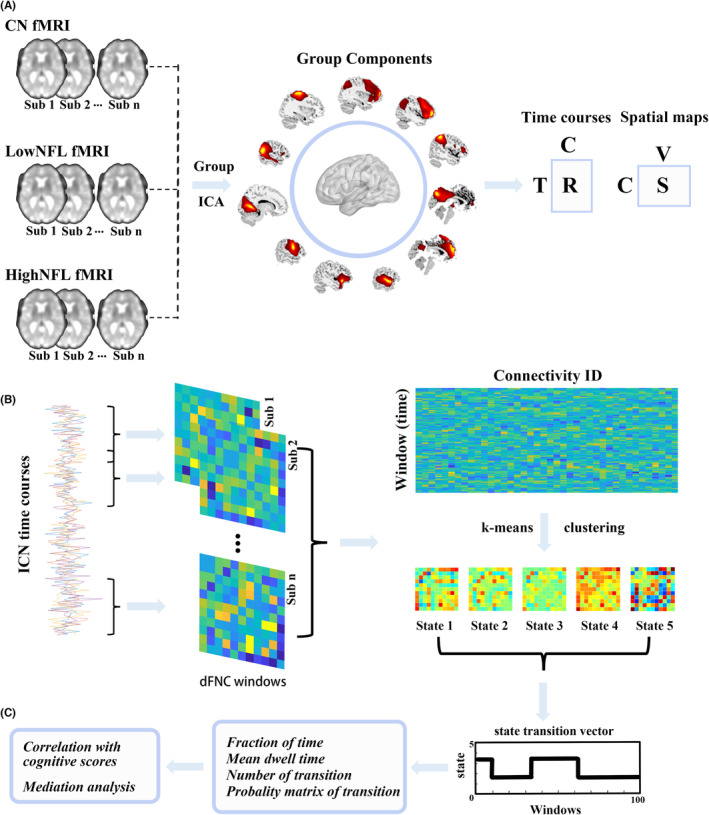
Pipeline of capturing whole‐brain connectivity features. (A) Group ICA is performed on three independent datasets, and the estimated independent components (ICs) are matched by spatial correlation. (B) Pearson correlation coefficients are calculated using the time courses across all scans, and then a sliding window approach is used to estimate dFNC. K‐means clustering is performed on the dFNC estimates. (C) State occurrences and transitions are calculated.

All image data analyzed here were obtained from the ADNI website (http://www.adni‐info.org). MRI scanners with a 3.0‐Tesla Philips were used for scanning all subjects. In this study, resting‐state fMRI was acquired using a gradient echo planar imaging sequence with the following parameters: repetition time (TR) = 3000 ms; echo time (TE) = 30 ms; slice thickness = 3.3 mm; flip angle = 80°; acquisition matrix = 64 × 64; slice number = 48; and spatial resolution = 3.31 × 3.31 × 3.31 mm^3^. Then, the data processing pipeline was divided into several main processing blocks, while the study flowchart is available in Figure 1.

### 2.6 rs‐fMRI preprocessing

The resting‐state fMRI data were preprocessed using the Data Processing Assistant for Resting‐State fMRI v2.2 (DPARSF) under the MATLAB R2013b environment. The following is a description of the data preprocessing procedure. A 10‐point time series was removed to lessen the impact of the scanner on the participants during their initial scanning and to facilitate their adaptation. Then, the functional images were slice time corrected for timing offsets between different slices and realigned to the first image to account for head motion between scans. Participants whose head motion exceeded 3 mm in translation and 3° in rotation were rejected from the study. Subsequently, spatial smoothing was performed using a Gaussian smoothing kernel with a full width at half maximum (FWHM) of 6 × 6 × 6 mm. After that, we applied a bandpass temporal filter (0.01–0.08 Hz). As a final step, nuisance covariates, including head motions, global mean signals, white matter signals, and cerebrospinal fluid signals, were removed from the regression calculations.

### 2.7 Group independent component analysis and postprocessing

Group‐level independent component analysis (ICA) for the preprocessed fMRI data was conducted with the Group ICA for fMRI Toolbox (GIFT version 4.0b).^28^, ^29^ First, data reduction was conducted to decrease computational complexity using a two‐stage principal component analysis (PCA). Specifically, the preprocessed fMRI data of both the patient and control groups were first dimensionally reduced in the temporal dimension, and then the dimensionality‐reduced data of all subjects were concatenated along the temporal dimension into a dataset, or grouped data, and reduced by another dimension. After that, the preprocessed data were decomposed into 40 independent components (ICs) through a two‐step PCA. To ensure the repeatability or stability of the decomposed independent components, we used the Infomax algorithm^30^ with 20 repetitions of ICASSO.^31^ Ultimately, using back reconstruction,^28^, ^32^ individual‐level components were transformed into Z scores, which represent how closely the time series of a given voxel matches the mean time series of that component. Then, we identified ICs of interest by using the automatic identification method and visual screening based on previously reported spatial maps. Of the 40 ICs obtained, 11 ICs of interest were determined that exhibited peak activation and higher low‐frequency spectral power predominantly in gray matter, with known spatial overlap with vessels, ventricles, white matter, and limbic regions. Eleven ICs were characterized as subnetworks: AN, DAN, DMN, left frontoparietal network (LFPN), right frontoparietal network (RFPN), SMN, ventral attention network (VAN), visual network (VN), and SN. In addition, following a previous study, we applied additional postprocessing steps on the time courses of the 11 ICs to eliminate noise, including (i) linear, quadratic, and cubic detrending; (ii) regression of their temporal; (iii) removal of detected outliers; and (iv) low‐pass filtering with a high‐frequency cut‐off of 0.15 Hz. Eventually, we performed the following dFNC analyses using the residual time courses.

### 2.8 Dynamic functional network connectivity

We adopted this analysis using the FNC toolbox in GIFT using the sliding window approach and k‐means clustering, which are common ways to estimate dFNC. The tapered windows were created by convolving a rectangle (window size set to 30 TRs) with a Gaussian of σ = 3 and slides in steps of 1 TR. Based on Pearson correlation, a covariance matrix was calculated to measure the dFNC between ICA time courses. Then, the k‐means clustering algorithm (using the squared Euclidean distance method with 500 iterations and 150 replicate dFNC windows) was conducted on the matrices. In addition, three dFNC indices were extracted from the target dFNC state of each subject, namely the fraction of time (FT), the mean dwell time (MDL), and the number of transitions (NT). FT refers to the percentage of the total time that occurs in each state. MDL indicates the average of time spent in a given state for each subject. NT reflects the number of times each participant switched between states. Subsequently, statistical analysis was performed using the nonparametric Whitney *U* test for these indicators (*p* < 0.05 was considered statistically significant).

Additionally, to determine whether plasma NFL mediated the relationships between dFNC indices and cognition, mediation analysis was further performed. Based on bootstrapping (*k* = 1000 random samples), bias‐corrected 95% confidence intervals (CIs) were calculated to test whether the mediation was significant. Statistical analyses were performed with PROCESS for the Statistical Package for Social Science (SPSS) version 22.0 for Windows (SPSS, Inc., Chicago, IL).

### 2.9 Graph theory analysis

We applied graph theory analysis using GRETNA software (www.nitrc.org/projects/gretna) to analyze the topological properties of the dynamic functional networks of each subject. Based on the previous graph theory framework, 11 ICs corresponded to functionally independent nodes and connectivities linking node pairs were defined as edges in the graphs. Then, all FNC matrixes were binarized, and a wide range of sparsity thresholds was set (threshold range of 0.05–0.40 with an interval of 0.01). At each sparsity threshold, global and local network efficiencies were employed to investigate local and global information in functional brain networks. Briefly, we calculated both global and nodal network properties and the area under the curve (AUC) for each property over the sparsity range and compared the AUC between each group. We applied variance to assess the differences in global and local efficiency.

### 2.10 Statistical analysis

The statistical analysis was performed with SPSS version 22.0 for Windows. The differences among CN, LowNFL, and HighNFL in demographic, neuropsychological, and plasma NFL were assessed by Kruskal–Wallis tests, a nonparametric test. Post hoc tests were also performed by another nonparametric test, the Mann–Whiney *U* test. We performed Spearman's correlation analysis to investigate the relationship between the dFNC characteristics and the neuropsychological assessment data in the HighNFL group. The statistical significance threshold was set at *p* < 0.05.

## 3 RESULTS

### 3.1 Demographic and neuropsychometric characteristics

Demographic and neuropsychometric characteristics are provided in Table 1. Comparisons of age, education level, or sex variables did not reveal any significant differences. General cognition and episodic memory (i.e., RAVLT_immediate, RAVLT_learning, and RAVLT_perc_forgetting) indicated significant differences between the CN and HighNFL groups, as well as the LowNFL and HighNFL groups. Furthermore, a previous study demonstrated that a significant correlation was found between NFL levels and cognitive impairment. In Table 1, we observed that plasma NFL levels were significantly higher in the HighNFL group than in the CN and LowNFL groups, and patients' general cognition (i.e., MMSE, MoCA, CDR, ADAS11/13, and FAQ) and episodic memory (i.e., RAVLT_immediate, RAVLT_learning, and RAVLT_perc_forgetting) worsened with plasma NFL level progression (*p* < 0.05).

**TABLE 1:** Demographic and neuropsychological data.

Items	CN (*n* = 29)	LowNFL (*n* = 55)	HighNFL (*n* = 55)	*p‐*Value	*p‐*(Mann–Whiney *U* test)		
					CN vs. LowNFL	CN vs. HighNFL	LowNFL vs. HighNFL
Demographics							
Age (years)	73.70 ± 4.92	70.32 ± 7.0	74.26 ± 6.04	0.057	0.053	0.374	0.143
Education (years)	15.79 ± 2.32	16.69 ± 2.43	15.22 ± 2.66	0.15	0.118	0.334	0.054
Gender (male/female)	1.62 ± 0.49	1.47 ± 0.5	1.55 ± 0.5	0.423	0.199	0.510	0.448
General cognition							
MMSE	29 ± 1.13	26.89 ± 3.2	24.18 ± 4.62	<0.001*	0.003*	<0.001*	<0.001*
MoCA	26.48 ± 2.35	21.91 ± 4.53	19.2 ± 5.55	<0.001*	<0.001*	<0.001*	0.003*
CDR	0.12 ± 0.26	2.34 ± 2.21	3.35 ± 2.41	<0.001*	<0.001*	<0.001*	0.010*
ADAS11	5.21 ± 2.70	12.18 ± 7.90	18.51 ± 11.61	<0.001*	<0.001*	<0.001*	0.001*
ADAS13	7.93 ± 4.37	18.20 ± 11.33	26.91 ± 14.83	<0.001*	<0.001*	<0.001*	<0.001*
FAQ	0.17 ± 0.66	5.67 ± 7.94	10.58 ± 9.07	<0.001*	<0.001*	<0.001*	0.005*
Episodic Memory							
RAVLT_immediate	46 ± 11.09	34.27 ± 12.64	27.05 ± 11.30	<0.001*	<0.001*	<0.001*	0.002*
RAVLT_learning	5.72 ± 2.72	4.07 ± 2.99	2.75 ± 2.24	<0.001*	0.013*	<0.001*	0.025*
RAVLT_forgetting	4.24 ± 2.61	4.62 ± 2.22	4.53 ± 1.94	0.486	0.274	0.280	0.897
RAVLT_perc_forgetting	41.32 ± 28.28	66.48 ± 34.15	80.06 ± 27.40	<0.001*	<0.001*	<0.001*	0.022*
Blood biomarker							
Plasma NFL	31.39 ± 7.79	31.79 ± 6.54	63.75 ± 24.56	<0.001*	0.655	<0.001*	<0.001*

*Note*: Values were presented as the average ± standard deviation (SD); *p‐*value: as the data do not satisfy the normal distribution, the *p‐*value was obtained by Kruskal–Wallis test. *p‐*(Mann–Whiney *U* Test) was used here due to the fact that the data were not normally distributed.

Abbreviations: ADAS, Alzheimer's Disease Assessment Scale–Cognitive section; CDR, Clinical Dementia Rating; CN, cognitively normal; FAQ, Functional activities questionnaire; HighNFL, high neurofilament light; LowNFL, low plasma neurofilament light; MMSE, Mini‐Mental State Examination; MoCA, Montreal Cognitive Assessment; Plasma NFL, Plasma neurofilament light chain; RAVLT, Rey Auditory Verbal Learning Test.

* Indicates a statistical difference between groups, *p* < 0.05.

### 3.2 ICs of interest

**FIGURE 2:**
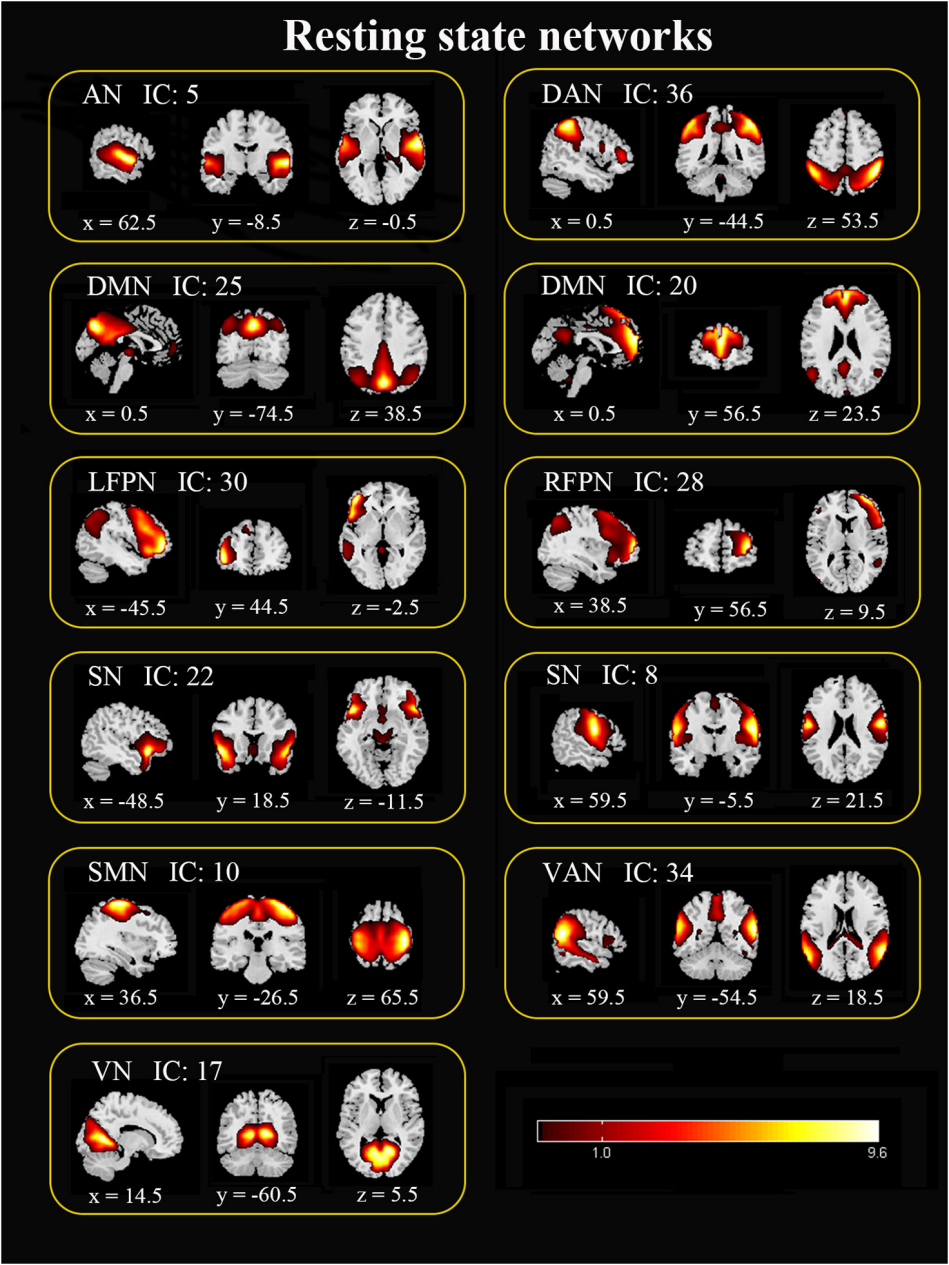
Eleven independent functional components were derived from the group ICA: auditory network (AN, IC 5), dorsal attention network (DAN, IC 36), default mode network (DMN, IC 25, 20), left frontoparietal network (LFPN, IC 30), right frontoparietal network (RFPN, IC 28), salience network (SN, IC 22, 8), somatomotor network (SMN, IC 10), ventral attention network (VAN, IC 34), and visual network (VN, IC 17).

Figure 2 shows the 11 ICs of interest, which were selected from the 40 ICs (one sample *t* test, *p* < 0.001, FDR corrected). Based on their anatomical and functional properties, 11 ICs were further categorized into nine networks, including the AN, DAN, DMN, LFPN, RFPN, SN, SMN, VAN, and VN.

### 3.3 dFNC states

**FIGURE 3:**
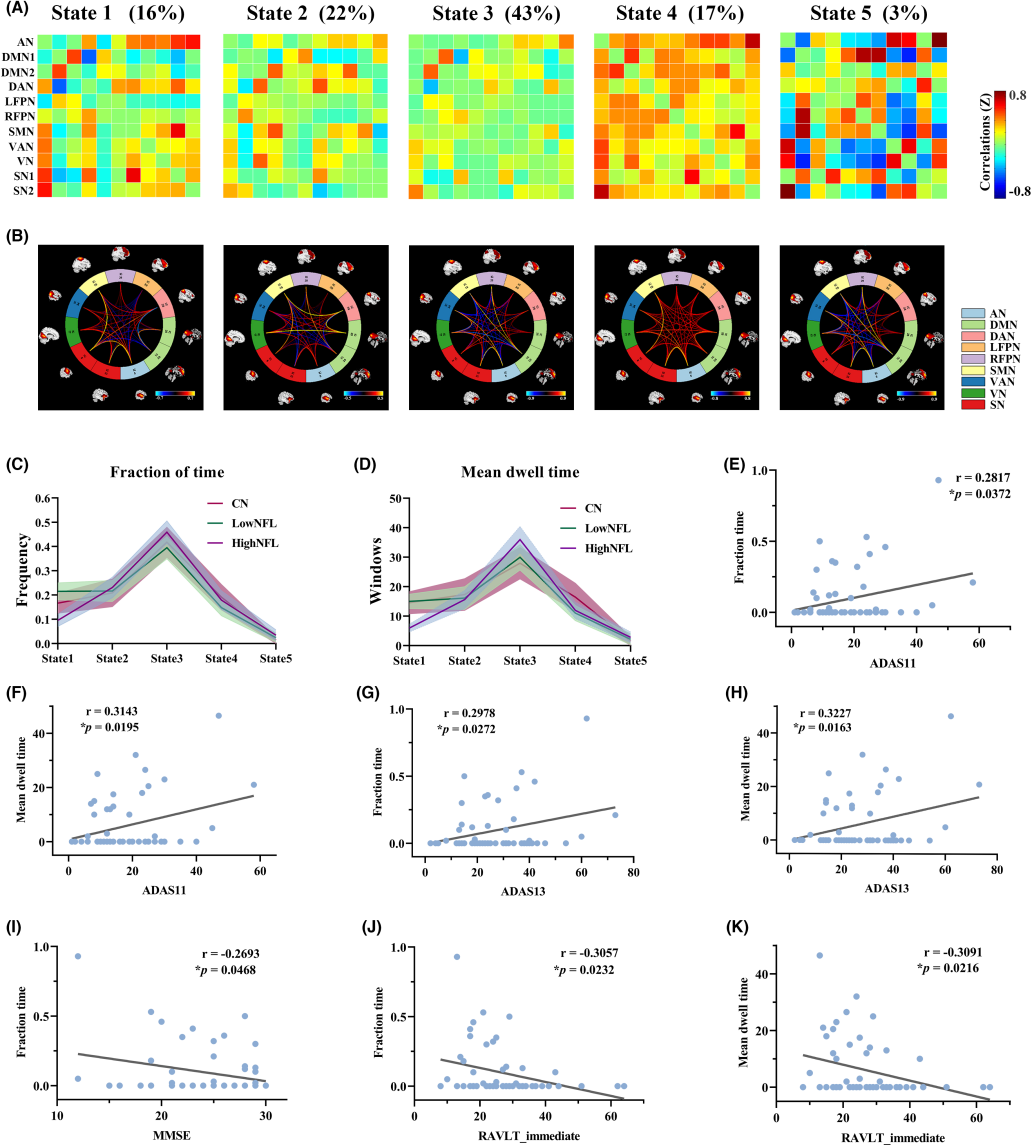
Dynamic functional connectivity state results. Identified dFNC states using the k‐means clustering method. (A) Cluster centroids of each state and percentage of occurrence of each brain connectivity state across the sliding windows of all subjects. The color bar shows the strength of the connectivity. (B) Visualization of functional network connectivity at every state. (C) Comparison of the group effect in the temporal properties of the dFNC state among the CN, LowNFL, and HighNFL groups (*p* < 0.05, FDR corrected): Fraction time. (D) Mean dwell time. (E–K) Correlations of the HighNFL group between cognitive scales and dFNC indices in state 1.

The sliding window approach was utilized to calculate the dFNC between ICs, and then the dFNC was estimated for all subjects using *k*‐means clustering to identify five states of connectivity that recurred over time. The five states of the clusters and their respective frequencies and percentages appear as in Figure 3A,B. Using the five clustering centroids (cluster medians) described above, all dFNC windows for each subject were classified into one of the five states based on the similarity of the starting clustering centroids. Note that not all subjects were assigned a dFNC window for each state; see the subject counts for each state shown in Table 2.

**TABLE 2:** Number of per group individuals in each state and their number of windowed FNC in parentheses.

Groups	N	State 1	State 2	State 3	State 4	State 5
CN	29	16	13	21	13	1
LowNFL	55	28	22	43	20	2
HighNFL	55	16	23	42	20	3
Total	139	60 (2193)	58 (3071)	106 (5912)	53 (2357)	6 (367)

*Note*: Number of individuals in per group participating in each state. The number of FNCs in the window is indicated in parentheses.

Abbreviation: N, number.

In state 1, which accounts for 16% of all windows, the FNC between the AN and SMN, VAN, VN, and SN showed positive connectivity and negative connectivity between the DMN and DAN and SN. In states 2 and 3, which accounted for 22% and 43% of all windows, the FNC between all functional networks was very sparse. Compared with state 2, state 4 showed the opposite connectivity pattern, such as a more positive correlation within networks.

With regard to temporal properties, the dFNC was computed in Figure 3C,D (i.e., fractional of time and mean dwell time). Figure S1A (for details, see Appendix S1) generated the state transition vector of three groups for 139 participants, as well as the number of transitions and matrix of transitions, as shown in Figure S1B,C (for details, see Appendix S1). Among these state‐related indicators in state 1, we found significant differences in fraction time and mean dwell time in the CN and HighNFL groups, as well as the LowNFL and HighNFL groups (Table 3). However, we did not observe any significant group differences in the other state‐related indicators. Furthermore, we also found that in state 1, the fraction of time and mean dwell time of the HighNFL group were lower than those of the CN and LowNFL groups.

**TABLE 3:** Group differences in temporal dynamic indices revealed by the states clustering analysis.

Indices	CN	LowNFL	HighNFL	*p‐*(Mann–Whiney *U* Test)		
				CN vs. LowNFL	CN vs. HighNFL	Low vs. HighNFL
FT in state 1	0.17 ± 0.23	0.22 ± 0.26	0.10 ± 0.19	0.564	0.048*	0.007*
FT in state 2	0.21 ± 0.31	0.22 ± 0.32	0.23 ± 0.31	0.912	0.888	0.875
FT in state 3	0.42 ± 0.33	0.40 ± 0.34	0.46 ± 0.35	0.751	0.624	0.325
FT in state 4	0.19 ± 0.28	0.15 ± 0.25	0.18 ± 0.29	0.387	0.875	0.509
FT in state 5	0.01 ± 0.08	0.02 ± 0.14	0.03 ± 0.16	0.524	0.924	0.416
MDL in state 1	14.57 ± 20.28	15.02 ± 18.37	5.96 ± 10.27	0.895	0.018*	0.005*
MDL in state 2	17.35 ± 28.91	16.14 ± 28.84	15.65 ± 23.62	0.748	0.952	0.722
MDL in state 3	27.98 ± 28.80	29.98 ± 32.18	36.0 ± 33.81	0.928	0.451	0.258
MDL in state 4	16.47 ± 25.44	11.06 ± 19.19	11.99 ± 21.37	0.342	0.593	0.632
MDL in state 5	1.48 ± 7.61	2.13 ± 13.64	2.83 ± 14.35	0.524	0.941	0.408
Transition Number	2.38 ± 1.68	2.55 ± 1.95	2.42 ± 1.64	0.834	0.893	0.913

*Note*: Values were presented as the average ± standard deviation (SD); *p‐*(Mann–Whiney *U* Test) was used here due to the fact that the data were not normally distributed.

Abbreviations: CN, cognitively normal; FT, fraction of time; HighNFL, high neurofilament light; LowNFL, low plasma neurofilament light; MDL, mean dwell time.

* Indicates a statistical difference between groups, *p* < 0.05.

### 3.4 Associations between dFNC features and cognitive scores

Figure 3E–K displays the results of a significant association between dFNC features and cognitive scores in the HighNFL group. In dFNC state 1, the fraction of time was positively correlated with ADAS11 (*r* = 0.2817, *p* = 0.0372) and ADAS13 (*r* = 0.2978, *p* = 0.0272), whereas it was negatively correlated with MMSE (*r* = −0.2693, *p* = 0.0468) and RAVLT_immediate (*r* = −0.3057, *p* = 0.0232). Similarly, we found that the mean dwell time was positively correlated with ADAS11 (*r* = 0.3143, *p* = 0.0195) and ADAS13 (*r* = 0.3227, *p* = 0.0163) and negatively correlated with RAVLT_immediate (*r* = −0.3091, *p* = 0.0216).

### 3.5 Graph topological properties

Graph theory analysis was applied to investigate the topologic metrics of dFNC states and compare them between groups (i.e., CN, LowNFL, and HighNFL). The variability of the global and local efficiency for the three groups is depicted in Figure S2 (for details, see Appendix S1). In global and local efficiency, we observed that LowNFL and HighNFL patients exhibited significantly higher global efficiency than CN (*p* < 0.05 FDR corrected), suggesting that average parallel information in brain networks of cognitively impaired patients can provide more efficient information transfer. In addition, we found a significant correlation between CN and LowNFL, as well as CN and HighNFL groups in local efficiency.

### 3.6 Mediators of plasma NFL

**FIGURE 4:**
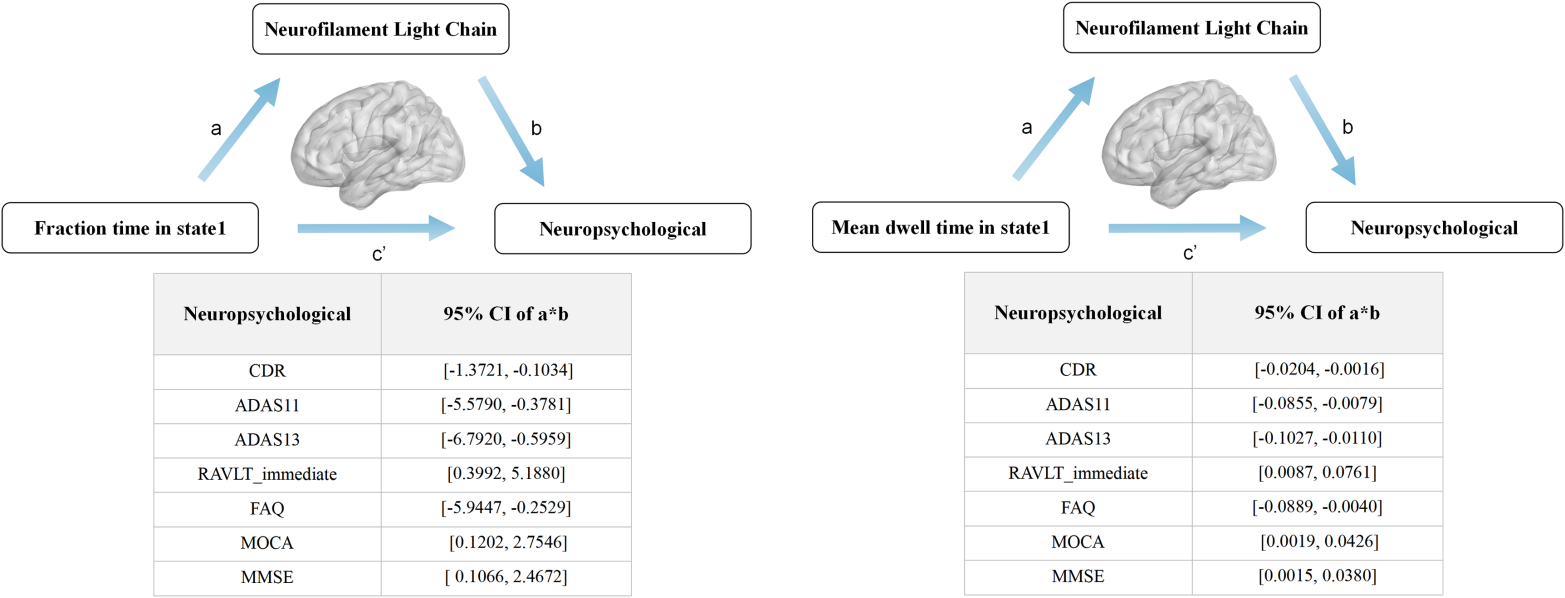
Relationships among plasma NFL, the temporal properties of the dFNC state, and cognition were revealed in cognitive impairment patients. Plasma NFL was used as a mediator, and fraction time affected general cognition and episodic memory by these mediators (*p* < 0.05). In addition, plasma NFL was used as a mediator, and mean dwell time affected general cognition and episodic memory by these mediators (*p* < 0.05).

Three parent‐reported variables (plasma NFL as a mediator, topologic metrics of dFNC states were entered as a predictor, and neuropsychological performance was an outcome) showed differential change by condition and were subjected to mediation analyses. In the cognitively impaired patients, plasma NFL was used as a mediator, and topologic metrics of dFNC states (i.e., fractional of time, FT; mean dwell time, MDL) affected general cognition and episodic memory by these mediators (*p* < 0.05, details see Figure 4). The results indicated that dynamic indicators influenced cognitive scale mainly through the mediator of plasma NFL in state 1. In detail, the effect of plasma NFL was found to be significant in the HighNFL group FT of CDR, ADAS11, ADAS13, RAVLT immediate, FAQ, MOCA, and MMSE (95% CIs in order were as follows: −1.3721, −0.1034; −5.5790, −0.3781; −6.7920, −0.5959; 0.3992, 5.1880; −5.9447, −0.2529; 0.1202, 2.7546; 0.1066, 2.4672, separately), while plasma NFL was found to be significant in the HighNFL group MDL of CDR, ADAS11, ADAS13, RAVLT immediate, FAQ, MOCA, and MMSE (95% CIs in order were as follows: −0.0204, −0.0016; −0.0855, −0.0079; −0.1027, −0.0110; 0.0087, 0.0761; −0.0889, −0.0040; 0.0019, 0.0426; 0.0015).

## 4 DISCUSSION

The present study explored the dynamics of whole‐brain FNC in CN, LowNFL, and HighNFL patients and emphasized the temporal properties of functional connectivity of dFNC states in relation to psychological scales. The main findings were as follows: (i) the dFNC in the 11 brain networks could be clustered into 5 states that recurred over time, which differed in their connectivity patterns, with 3 states showing dense connectivity (states 1, 4, and 5) and 2 states showing sparse connectivity (states 2 and 3); (ii) analysis of the temporal properties of the functional connectivity of the dFNC states revealed that the HighNFL group spent less time in state 1 than controls and had significantly lower fraction time and mean dwell time, but the opposite in state 3; and (iii) a potential mechanism for the association between dFNC indicators and plasma NFL levels in cognitively impaired patients, in addition, it could be used to distinguish normal patients from cognitively impaired patients. These results suggested that the dynamic behavior of brain connections should be highlighted as AD related.

Transient dFNC states may reveal the functional capacity of the nervous system.^33^ Strengthened integration between networks is necessary for greater working memory performance.^34^, ^35^ A recent study showed that the motor network in AD subjects is independent of other brain networks and is in a sparsely connected functional connectivity state most of the time.^36^ In addition, another study demonstrated that the time spent by AD patients differed when they were in different states of connectivity. Namely, states with lower connectivity spend more time, while states with higher connectivity spend less time.^37^ Our results showed that the subjects in the HighNFL group spent more time in state 3, which showed sparse connectivity between brain networks, and less time in the state with stronger connectivity between brain networks. Next, another part of the results was identified by showing that the normal brain spends more time in tight junctions than HighNFL, as in state 1. Therefore, this reinforces the role of connectivity sparseness and denseness in the transition from the normal to the cognitive impairment stage in different states.

Previous work has demonstrated the overlap between areas of pathological damage in AD and regions of the DMN.^38^ Notably, activation in the DMN plays a significant role in goal‐directed and introspective cognitive control associated with episodic memory.^39^, ^40^ Therefore, as prior studies have demonstrated, we expected the role of the DMN as a pivotal brain function to be compromised. However, we found that the strength of the functional connectivity between the SMN/SN was most associated with symptom severity. Meanwhile, the DMN was tightly connected to internal functions in four states but sparsely connected to other brain networks. In the HighNFL group, the time spent in state 1 was positively correlated with the ADAS11/13 score but negatively correlated with the ADAS11/13 score. The SMN and SN are assumed to be less affected by AD pathology in the later stages of cognitive impairment. Therefore, these stimulating findings may serve as a measure of sensitivity for monitoring neurological damage in AD (possibly more sensitive than regions of primary degeneration). Therefore, we speculated that SMN and SN may impact cognitive function by dynamically regulating functional connections within the networks. Additionally, our results may suggest that the DMN is an important brain network affected by AD progression to later stages, which could also help explain why changes in motor function may occur preferentially and earlier than the onset of cognition and dementia.^41^ Large, population‐based prospective studies are needed to identify more precise changes in sensory or motor function that mark early cognitive impairment.

NFL is one of the three subunits of neurofilament proteins in the central nervous system, which are essential cytoskeletal proteins for neurons and are abundantly present in most myelinated axons.^42^ NFL is a dynamic biomarker that is released into the circulation and eventually into the bloodstream following axonal injury. NFL levels have been associated with neurodegenerative diseases, such as AD.^5^ NFL levels were positively correlated with cerebral axonal degeneration, and the higher the NFL, the more severe the cerebral axonal degeneration.^43^ Clinical studies have also indicated that plasma NFL is higher in patients who suffer from MCI or AD dementia with pathological features Aβ.^43^ Previous studies have also shown that age affects plasma NFL levels.^44^ Previous studies from our research team demonstrated that plasma NFL could affect the interactions of the core subsystem and FPN, which leads to cognitive decline in AD spectrum patients.^45^ Some studies have addressed the relationship between plasma NFL and structural and functional changes in the brain. For example, in cognitively impaired subjects, plasma NFL was significantly associated with material atrophy in the temporal lobe and anterior and posterior cingulate. Meanwhile, it can also independently predict hippocampal atrophy.^46^ This study further highlighted the effect of plasma NFL on the temporal properties of dFNC states in relation to psychological scales.

This study had several limitations. First, given the relatively small sample size of our study population and based on a heterogeneous group, it prevented us from a comprehensive assessment. Future studies with larger sample sizes or multicenter clinical studies are recommended to validate this result and to further evaluate the impact of disease heterogeneity on dFNC. Second, the analytical approach of dFNC is relatively new and in this case, lacks a gold standard. For example, there is no common standard for how parameters such as the optimal window length and overlap should be chosen, whereas variations in these parameters may have a large impact on the analytical results.^47^ The choice of window size significantly affects the clustering. A window that is considered longer does not capture the true dynamic behavior, but more smoothing could be accomplished, while a shorter window can detect faster fluctuations. Future work could be completed to evaluate the variation in state derivatives over a range of window sizes.^48^ There was only one value of the plasma NFL index in this study, and the relationship between NFL over time and dFNC can be studied in a follow‐up.

## 5 CONCLUSIONS

In summary, our study examined common and specific dFNC abnormalities in the brain networks of cognitive impairment patients at different levels of the inflammation‐related indicator NFL. We found that the HighNFL group preferred to spend less time in the dense connection state and spent more time in the sparse connection state. Moreover, our findings suggested decreased information processing and cognitive abilities in the HighNFL group, which may contribute to our clinical understanding of their abnormalities in emotional and cognitive functions. In brief, characteristic changes in the inflammation‐coupled dynamic brain network may provide alternative biomarkers for the assessment of disease severity of cognitive impairment.

## AUTHOR CONTRIBUTIONS

All authors contributed to the study's conception and design. YW: performed data analysis and wrote the manuscript. ZH and ZX were responsible for literature research. BF performed funding acquisition, study concept, and study design. YW, ZH, ZX, CH, and BF performed editing and approval of the manuscript.

## FUNDING INFORMATION

This work was supported partly by grants from the National Natural Science Foundation of China (No. 82071186), Clinical Trials from the Affiliated Drum Tower Hospital, Medical School of Nanjing University (No. 2022‐LCYG‐MS‐05), National Key Research and Development Program of China (No. 2022YFA1105300), and Jiangsu Province Senior Health Project (No. LKZ2023014).

## CONFLICT OF INTEREST STATEMENT

The authors declare that they have no competing interests.

## Supporting information


Appendix S1

## Data Availability

The original contributions presented in the study are included in the article/Appendix S1, and further inquiries can be directed to the corresponding authors.

## References

[1] Scheltens P , De Strooper B , Kivipelto M , et al. Alzheimer's disease. Lancet (London, England). 2021;397(10284):1577‐1590. 33667416 10.1016/S0140-6736(20)32205-4 PMC8354300

[2] Alzheimer's disease facts and figures. Alzheimers Dement. 2020;17:327‐406. 10.1002/alz.12328 33756057

[3] Hodson R . Alzheimer's disease. Nature. 2018;559(7715):S1. 30046078 10.1038/d41586-018-05717-6

[4] Jiang R , Calhoun VD , Zuo N , et al. Connectome‐based individualized prediction of temperament trait scores. Neuroimage. 2018;183:366‐374. 30125712 10.1016/j.neuroimage.2018.08.038

[5] Mattsson N , Andreasson U , Zetterberg H , Blennow K . Association of Plasma Neurofilament Light with Neurodegeneration in patients with Alzheimer disease. JAMA Neurol. 2017;74(5):557‐566. 28346578 10.1001/jamaneurol.2016.6117 PMC5822204

[6] Gisslén M , Price RW , Andreasson U , et al. Plasma concentration of the Neurofilament light protein (NFL) is a biomarker of CNS injury in HIV infection: a cross‐sectional study. EBioMedicine. 2016;3:135‐140. 26870824 10.1016/j.ebiom.2015.11.036 PMC4739412

[7] Disanto G , Barro C , Benkert P , et al. Serum Neurofilament light: a biomarker of neuronal damage in multiple sclerosis. Ann Neurol. 2017;81(6):857‐870. 28512753 10.1002/ana.24954 PMC5519945

[8] Zetterberg H , Skillbäck T , Mattsson N , et al. Association of Cerebrospinal Fluid Neurofilament Light Concentration with Alzheimer Disease Progression. JAMA Neurol. 2016;73(1):60‐67. 26524180 10.1001/jamaneurol.2015.3037 PMC5624219

[9] Hansson O , Janelidze S , Hall S , et al. Blood‐based NfL: a biomarker for differential diagnosis of parkinsonian disorder. Neurology. 2017;88(10):930‐937. 28179466 10.1212/WNL.0000000000003680 PMC5333515

[10] Friston KJ . Functional and effective connectivity: a review. Brain Connect. 2011;1(1):13‐36. 22432952 10.1089/brain.2011.0008

[11] Sporns O . From simple graphs to the connectome: networks in neuroimaging. Neuroimage. 2012;62(2):881‐886. 21964480 10.1016/j.neuroimage.2011.08.085

[12] Wang K , Liang M , Wang L , et al. Altered functional connectivity in early Alzheimer's disease: a resting‐state fMRI study. Hum Brain Mapp. 2007;28(10):967‐978. 17133390 10.1002/hbm.20324 PMC6871392

[13] Damoiseaux JS , Prater KE , Miller BL , Greicius MD . Functional connectivity tracks clinical deterioration in Alzheimer's disease. Neurobiol Aging. 2012;33(4):828.e819‐30. 10.1016/j.neurobiolaging.2011.06.024 PMC3218226 21840627

[14] Myers N , Pasquini L , Göttler J , et al. Within‐patient correspondence of amyloid‐β and intrinsic network connectivity in Alzheimer's disease. Brain. 2014;137(Pt 7):2052‐2064. 24771519 10.1093/brain/awu103 PMC4065018

[15] Wang P , Zhou B , Yao H , et al. Aberrant intra‐ and internetwork connectivity architectures in Alzheimer's disease and mild cognitive impairment. Sci Rep. 2015;5:14824. 26439278 10.1038/srep14824 PMC4594099

[16] Ćurčić‐Blake B , Ford JM , Hubl D , et al. Interaction of language, auditory and memory brain networks in auditory verbal hallucinations. Prog Neurobiol. 2017;148:1‐20. 27890810 10.1016/j.pneurobio.2016.11.002 PMC5240789

[17] Cai J , Liu A , Mi T , et al. Dynamic graph theoretical analysis of functional connectivity in Parkinson's disease: the importance of Fiedler value. IEEE J Biomed Health Inform. 2019;23(4):1720‐1729. 30307882 10.1109/JBHI.2018.2875456

[18] Liu F , Wang Y , Li M , et al. Dynamic functional network connectivity in idiopathic generalized epilepsy with generalized tonic–clonic seizure. Hum Brain Mapp. 2017;38:957‐973. 27726245 10.1002/hbm.23430 PMC6866949

[19] Xue K , Liang S , Yang B , et al. Local dynamic spontaneous brain activity changes in first‐episode, treatment‐naïve patients with major depressive disorder and their associated gene expression profiles. Psychol Med. 2022;52:2052‐2061. 33121546 10.1017/S0033291720003876

[20] Sendi MSE , Zendehrouh E , Miller RL , et al. Alzheimer's disease projection from Normal to mild dementia reflected in functional network connectivity: a longitudinal study. Frontiers in Neural Circuits. 2020;14:593263. 33551754 10.3389/fncir.2020.593263 PMC7859281

[21] Fu Z , Caprihan A , Chen J , et al. Altered static and dynamic functional network connectivity in Alzheimer's disease and subcortical ischemic vascular disease: shared and specific brain connectivity abnormalities. Hum Brain Mapp. 2019;40(11):3203‐3221. 30950567 10.1002/hbm.24591 PMC6865624

[22] De Scheerder MA , Van Hecke C , Zetterberg H , et al. Evaluating predictive markers for viral rebound and safety assessment in blood and lumbar fluid during HIV‐1 treatment interruption. J Antimicrob Chemother. 2020;75(5):1311‐1320. 32053203 10.1093/jac/dkaa003 PMC7869780

[23] Khalil M , Teunissen CE , Otto M , et al. Neurofilaments as biomarkers in neurological disorders. Nat Rev Neurol. 2018;14(10):577‐589. 30171200 10.1038/s41582-018-0058-z

[24] Peters N , van Leijsen E , Tuladhar AM , et al. Serum Neurofilament light chain is associated with incident lacunes in progressive cerebral small vessel disease. J Stroke. 2020;22(3):369‐376. 33053952 10.5853/jos.2019.02845 PMC7568975

[25] Preische O , Schultz SA , Apel A , et al. Serum neurofilament dynamics predicts neurodegeneration and clinical progression in presymptomatic Alzheimer's disease. Nat Med. 2019;25(2):277‐283. 30664784 10.1038/s41591-018-0304-3 PMC6367005

[26] Mielke MM , Syrjanen JA , Blennow K , et al. Plasma and CSF neurofilament light: relation to longitudinal neuroimaging and cognitive measures. Neurology. 2019;93(3):e252‐e260. 31182505 10.1212/WNL.0000000000007767 PMC6656645

[27] Benedet AL , Leuzy A , Pascoal TA , et al. Stage‐specific links between plasma neurofilament light and imaging biomarkers of Alzheimer's disease. Brain. 2020;143(12):3793‐3804. 33210117 10.1093/brain/awaa342 PMC7805809

[28] Calhoun VD , Adali T , Pearlson GD , Pekar JJ . A method for making group inferences from functional MRI data using independent component analysis. Hum Brain Mapp. 2001;14(3):140‐151. 11559959 10.1002/hbm.1048 PMC6871952

[29] Calhoun V . Group ICA of fMRI toolbox (GIFT). 2004 Online at http://icatb.sourceforge.net

[30] Bell AJ , Sejnowski TJ . An information‐maximization approach to blind separation and blind deconvolution. Neural Comput. 1995;7(6):1129‐1159. 7584893 10.1162/neco.1995.7.6.1129

[31] Himberg J , Hyva¨rinen A . Icasso: software for investigating the reliability of ICA estimates by clustering and visualization. Neural Networks for Signal Processing, 2003. NNSP′03 IEEE 13th workshop; 2003:259‐268.

[32] Erhardt EB , Rachakonda S , Bedrick EJ , Allen EA , Adali T , Calhoun VD . Comparison of multisubject ICA methods for analysis of fMRI data. Hum Brain Mapp. 2011;32(12):2075‐2095. 21162045 10.1002/hbm.21170 PMC3117074

[33] Deco G , Jirsa VK , McIntosh AR . Emerging concepts for the dynamical organization of resting‐state activity in the brain. Nat Rev Neurosci. 2011;12(1):43‐56. 21170073 10.1038/nrn2961

[34] Cohen JR , D'Esposito M . The segregation and integration of distinct brain networks and their relationship to cognition. J Neurosci. 2016;36(48):12083‐12094. 27903719 10.1523/JNEUROSCI.2965-15.2016 PMC5148214

[35] Kitzbichler MG , Henson RN , Smith ML , Nathan PJ , Bullmore ET . Cognitive effort drives workspace configuration of human brain functional networks. J Neurosci. 2011;31(22):8259‐8270. 21632947 10.1523/JNEUROSCI.0440-11.2011 PMC6622866

[36] Schumacher J , Peraza LR , Firbank M , et al. Dynamic functional connectivity changes in dementia with Lewy bodies and Alzheimer's disease. Neuroimage Clinical. 2019;22:101812. 30991620 10.1016/j.nicl.2019.101812 PMC6462776

[37] Du Y , Fu Z , Sui J , et al. NeuroMark: an adaptive independent component analysis framework for estimating reproducible and comparable fMRI biomarkers among brain disorders. medRxiv (Preprint). 2019. doi:10.1101/19008631

[38] Mevel K , Chételat G , Eustache F , Desgranges B . The default mode network in healthy aging and Alzheimer's disease. Int J Alzheimers Dis. 2011;2011:535816. 21760988 10.4061/2011/535816 PMC3132539

[39] Yamashita M , Shimokawa T , Peper F , Tanemura R . Functional network activity during errorless and trial‐and‐error color‐name association learning. Brain Behav. 2020;10(8):e01723. 32558312 10.1002/brb3.1723 PMC7428483

[40] Spreng RN , Stevens WD , Chamberlain JP , Gilmore AW , Schacter DL . Default network activity, coupled with the frontoparietal control network, supports goal‐directed cognition. Neuroimage. 2010;53(1):303‐317. 20600998 10.1016/j.neuroimage.2010.06.016 PMC2914129

[41] Wesson DW , Borkowski AH , Landreth GE , Nixon RA , Levy E , Wilson DA . Sensory network dysfunction, behavioral impairments, and their reversibility in an Alzheimer's β‐amyloidosis mouse model. J Neurosci. 2011;31(44):15962‐15971. 22049439 10.1523/JNEUROSCI.2085-11.2011 PMC3417321

[42] Ashton NJ , Janelidze S , Al Khleifat A , et al. A multicentre validation study of the diagnostic value of plasma neurofilament light. Nat Commun. 2021;12(1):3400. 34099648 10.1038/s41467-021-23620-z PMC8185001

[43] Bacioglu M , Maia LF , Preische O , et al. Neurofilament light chain in blood and CSF as marker of disease progression in mouse models and in neurodegenerative diseases. Neuron. 2016;91(2):494‐496. 27477021 10.1016/j.neuron.2016.07.007

[44] Lin YS , Lee WJ , Wang SJ , Fuh JL . Levels of plasma neurofilament light chain and cognitive function in patients with Alzheimer or Parkinson disease. Sci Rep. 2018;8(1):17368. 30478269 10.1038/s41598-018-35766-w PMC6255914

[45] Yao W , Zhang X , Zhao H , Xu Y , Bai F . Inflammation disrupts cognitive integrity via plasma Neurofilament light chain coupling brain networks in Alzheimer's disease. J Alzheimers Dis. 2022;89(2):505‐518. 35871350 10.3233/JAD-220475

[46] Mattsson N , Cullen NC , Andreasson U , Zetterberg H , Blennow K . Association between longitudinal plasma Neurofilament light and neurodegeneration in patients with Alzheimer disease. JAMA Neurol. 2019;76(7):791‐799. 31009028 10.1001/jamaneurol.2019.0765 PMC6583067

[47] Shakil S , Lee CH , Keilholz SD . Evaluation of sliding window correlation performance for characterizing dynamic functional connectivity and brain states. Neuroimage. 2016;133:111‐128. 26952197 10.1016/j.neuroimage.2016.02.074 PMC4889509

[48] Faghiri A , Iraji A , Damaraju E , Turner J , Calhoun VD . A unified approach for characterizing static/dynamic connectivity frequency profiles using filter banks. Netw Neurosci (Cambridge, Mass). 2021;5(1):56‐82. 10.1162/netn_a_00155 PMC7935048 33688606

