# Supplementary material for: Inflammation affects dynamic functional network connectivity pattern changes via plasma NFL in cognitive impairment patients
Source: CNS Neurosci Ther. 2023 Aug 7;30(2):e14391. doi: 10.1111/cns.14391 (PMC10848064; doi:10.1111/cns.14391)

**Supporting Information**

**Inflammation affects dynamic functional network connectivity pattern changes via plasma NFL in cognitive impairment patients**

Weina Yao^1^, Huijuan Zhou^2^, Xiao Zhang^2^, Haifeng Chen^2^, Feng Bai^3,4,5^* and Alzheimer’s Disease Neuroimaging Initiative^a^

^1^Department of Neurology, Taikang Xianlin Drum Tower Hospital Clinical College of Wuhan University, Nanjing 210008, China.

^2^Department of Neurology, Nanjing Drum Tower Hospital Clinical College of Traditional Chinese and Western Medicine, Nanjing University of Chinese Medicine, Nanjing 210008, China.

^3^Geriatric Medicine Center, Taikang Xianlin Drum Tower Hospital Clinical College of Wuhan University, Nanjing 210008, China.

^4^Geriatric Medicine Center, Taikang Xianlin Drum Tower Hospital, Affiliated Hospital of Medical School, Nanjing University, Nanjing 210008, China.

^5^Department of Neurology, Nanjing Drum Tower Hospital, Affiliated Hospital of Medical School, Nanjing University, Nanjing 210008, China.

*** Corresponding author**

Feng Bai

E-mail address: [baifeng515@126.com](mailto:baifeng515@126.com)

^a^Data used in preparation of this article were obtained from the Alzheimer’s Disease Neuroimaging Initiative (ADNI) database (adni.loni.usc.edu). As such, the investigators within the ADNI contributed to the design and implementation of the ADNI and/or provided data but did not participate in the analysis or writing of this report. A complete listing of ADNI investigators can be found at <http://adni.loni.usc.edu/wp-content/uploads/how_to_apply/ADNI_Acknowledgement_List.pdf>.

**SI part I. Inclusion Criteria and Exclusion Criteria**

***Inclusion Criteria***

**(1) Cognitively normal**

1. The subject must be free of memory complaints, verified by a study partner, beyond what one would expect for age.

2. Normal memory function documented by scoring above education-adjusted cut-offs on the Logical Memory II subscale (Delayed Paragraph Recall, Paragraph A only) from the Wechsler Memory Scale–Revised (the maximum score is 25): a. ≥9 for 16 or more years of education. b. ≥5 for 8-15 years of education. c. ≥3 for 0-7 years of education.

3. Mini-Mental State Exam score between 24 and 30 (inclusive). (Exceptions may be made for subjects with less than 8 years of education at the discretion of the project director).

4. Clinical Dementia Rating=0. The memory box score must be 0.

5. Cognitively normal, based on an absence of significant impairment in cognitive functions or activities of daily living.

6. Stability of Permitted Medications for 4 weeks. In particular, subjects may a. take stable doses of antidepressants lacking significant anticholinergic side effects (if they are not currently depressed and do not have a history of major depression within the past 1 year). b. Estrogen replacement therapy is permissible. c. Gingko biloba is permissible but discouraged, d. Washout from psychoactive medication (e.g., excluded antidepressants, neuroleptics, chronic anxiolytics or sedative hypnotics, etc.) for at least 4 weeks prior to screening.

**(2) EMCI**

1. The subject must have a subjective memory concern as reported by the subject, study partner, or clinician.

2. Abnormal memory function documented by scoring within the education-adjusted ranges on the Logical Memory II subscale (Delayed Paragraph Recall, Paragraph A only) from the Wechsler Memory Scale –Revised (the maximum score is 25): a. 9-11 for 16 or more years of education. b. 5-9 for 8-15 years of education. c. 3-6 for 0-7 years of education.

3. Mini-Mental State Exam score between 24 and 30 (inclusive). (Exceptions may be made for subjects with less than 8 years of education at the discretion of the project director).

4. Clinical Dementia Rating=0.5. The memory box score must be at least 0.5.

5. General cognition and functional performance were sufficiently preserved such that a diagnosis of Alzheimer's disease could not be made by the site physician at the time of the screening visit.

6. Stability of Permitted Medications for 4 weeks. In particular, subjects may a. take stable doses of antidepressants lacking significant anticholinergic side effects (if they are not currently depressed and do not have a history of major depression within the past 1 year). b. Estrogen replacement therapy is permissible. c. Gingko biloba is permissible but discouraged. d. Washout from psychoactive medication (e.g., excluded antidepressants, neuroleptics, chronic anxiolytics or sedative hypnotics, etc.) for at least 4 weeks prior to screening. e. Cholinesterase inhibitors and memantine are allowable if stable for 12 weeks prior to screening.

**(3) LMCI**

1. The subject must have a subjective memory concern as reported by the subject, study partner, or clinician.

2. Abnormal memory function documented by scoring within the education-adjusted ranges on the Logical Memory II subscale (Delayed Paragraph Recall, Paragraph A only) from the Wechsler Memory Scale –Revised (the maximum score is 25): a. ≤8 for 16 or more years of education. b. ≤4 for 8-15 years of education. c. ≤2 for 0-7 years of education.

3. Mini-Mental State Exam score between 24 and 30 (inclusive). (Exceptions may be made for subjects with less than 8 years of education at the discretion of the project director).

4. Clinical Dementia Rating=0.5. The memory box score must be at least 0.5.

5. General cognition and functional performance were sufficiently preserved such that a diagnosis of Alzheimer's disease could not be made by the site physician at the time of the screening visit.

6. Stability of Permitted Medications for 4 weeks. In particular, subjects may a. take stable doses of antidepressants lacking significant anticholinergic side effects (if they are not currently depressed and do not have a history of major depression within the past 1 year). b. Estrogen replacement therapy is permissible. c. Gingko biloba is permissible but discouraged. d. Washout from psychoactive medication (e.g., excluded antidepressants, neuroleptics, chronic anxiolytics or sedative hypnotics, etc.) for at least 4 weeks prior to screening. e. Cholinesterase inhibitors and memantine are allowable if stable for 12 weeks prior to screening.

**(4) AD**

1. The subject must have a subjective memory concern as reported by the subject, study partner, or clinician.

2. Abnormal memory function documented by scoring within the education-adjusted ranges on the Logical Memory II subscale (Delayed Paragraph Recall, Paragraph A only) from the Wechsler Memory Scale –Revised (the maximum score is 25): a. ≤8 for 16 or more years of education, b. ≤4 for 8-15 years of education, c. ≤2 for 0-7 years of education.

3. Mini-Mental State Exam score between 20 and 26 (inclusive) (exceptions may be made for subjects with less than 8 years of education at the discretion of the project director).

4. Clinical Dementia Rating=0.5 or 1.0.

5. NINCDS/ADRDA criteria for probable AD.

6. Stability of Permitted Medications for 4 weeks. In particular, subjects may a. take stable doses of antidepressants lacking significant anticholinergic side effects (if they are not currently depressed and do not have a history of major depression within the past 1 year). b. Estrogen replacement therapy is permissible. c. Gingko biloba is permissible but discouraged. d. Washout from psychoactive medication (e.g., excluded antidepressants, neuroleptics, chronic anxiolytics or sedative hypnotics, etc.) for at least 4 weeks prior to screening. e. Cholinesterase inhibitors and memantine are allowable if stable for 12 weeks prior to screening.

**(5) Additional Inclusion Criteria: All Diagnostic Categories (Cognitively normal, EMCI, LMCI and AD)**

7. Geriatric Depression Scale less than 6.

8. Age between 55-90 (inclusive).

9. Study partners are available who have frequent contact with the subject (e.g., an average of 10 hours per week or more) and can accompany the subject to all clinic visits for the duration of the protocol.

10. Visual and auditory acuity adequate for neuropsychological testing.

11. Good general health with no diseases expected to interfere with the study.

12. Participant is not pregnant, lactating, or of childbearing potential (i.e., women must be two years postmenopausal or surgically sterile).

13. Willing and able to participate in a longitudinal imaging study.

14. Hachinski less than or equal to 4.

15. Completed six grades of education or had a good work history (sufficient to exclude mental retardation).

16. Must speak English or Spanish fluently.

17. Willing to undergo repeated MRIs (3Tesla) and at least two PET scans (one FDG and one amyloid imaging) and no medical contraindications to MRI.

18. Agrees to collection of blood for GWAS, APOE testing and DNA and RNA banking.

19. Agrees to collection of blood for biomarker testing.

20. Agrees to at least one lumbar puncture for the collection of CSF.

***Exclusion Criteria***

**(1) Cognitively normal**

1. Any significant neurologic disease, such as Parkinson's disease, multi-infarct dementia, Huntington's disease, normal pressure hydrocephalus, brain tumor, progressive supranuclear palsy, seizure disorder, subdural hematoma, multiple sclerosis, or history of significant head trauma followed by persistent neurologic defaults or known structural brain abnormalities.

**(2) EMCI**

1. Any significant neurologic disease other than suspected incipient Alzheimer's disease, such as Parkinson's disease, multi-infarct dementia, Huntington's disease, normal pressure hydrocephalus, brain tumor, progressive supranuclear palsy, seizure disorder, subdural hematoma, multiple sclerosis, or history of significant head trauma followed by persistent neurologic defaults or known structural brain abnormalities.

**(3) LMCI**

1. Any significant neurologic disease other than suspected incipient Alzheimer's disease, such as Parkinson's disease, multi-infarct dementia, Huntington's disease, normal pressure hydrocephalus, brain tumor, progressive supranuclear palsy, seizure disorder, subdural hematoma, multiple sclerosis, or history of significant head trauma followed by persistent neurologic defaults or known structural brain abnormalities.

**(4) AD**

1. Any significant neurologic disease other than Alzheimer's disease, such as Parkinson's disease, multi-infarct dementia, Huntington's disease, normal pressure hydrocephalus, brain tumor, progressive supranuclear palsy, seizure disorder, subdural hematoma, multiple sclerosis, or history of significant head trauma followed by persistent neurologic defaults or known structural brain abnormalities.

**(5) Additional exclusion criteria: All diagnostic categories**

2. Screening/baseline MRI scan with evidence of infection, infarction, or other focal lesions. Participants with multiple lacunes or lacunes in a critical memory structure were excluded.

3. Presence of pacemakers, aneurysm clips, artificial heart valves, ear implants, metal fragments or foreign objects in the eyes, skin or body.

4. Major depression, bipolar disorder as described in DSM-IV within the past 1 year. Psychotic features, agitation or behavioral problems within the last 3 months that could lead to difficulty complying with the protocol.

5. History of schizophrenia (DSM IV criteria).

6. History of alcohol or substance abuse or dependence within the past 2 years (DSM IV criteria).

7. Any significant systemic illness or unstable medical condition that could lead to difficulty complying with the protocol.

8. Clinically significant abnormalities in B12 or TFTs that might interfere with the study. A low B12 is exclusionary unless follow-up labs (homocysteine (HC) and methylmalonic acid (MMA)) indicate that it is not physiologically significant.

9. Residence in a skilled nursing facility.

10. Current use of specific psychoactive medications (e.g., certain antidepressants, neuroleptics, chronic anxiolytics or sedative hypnotics). Current use of warfarin (exclusionary for lumbar puncture).

11. Investigational agents are prohibited one month prior to entry and for the duration of the trial.

12. Participation in clinical studies involving neuropsychological measures being collected more than once per year.

13. Exceptions to these guidelines may be considered on a case-by-case basis at the discretion of the protocol director (Dr. Petersen).

**SI part II. Neuroimaging data**

**Figure S1.** (A) Brain states. The three groups showed five states. Color denotes distinct states in each subject. (B) Comparison of the group effect in the temporal properties of the dFNC state among the CN, LowNFL, and HighNFL groups (p < 0.05, FDR corrected): number of transitions. (C) The state transition probability matrix, averaged over subjects. High values indicate a high probability of staying in a state.


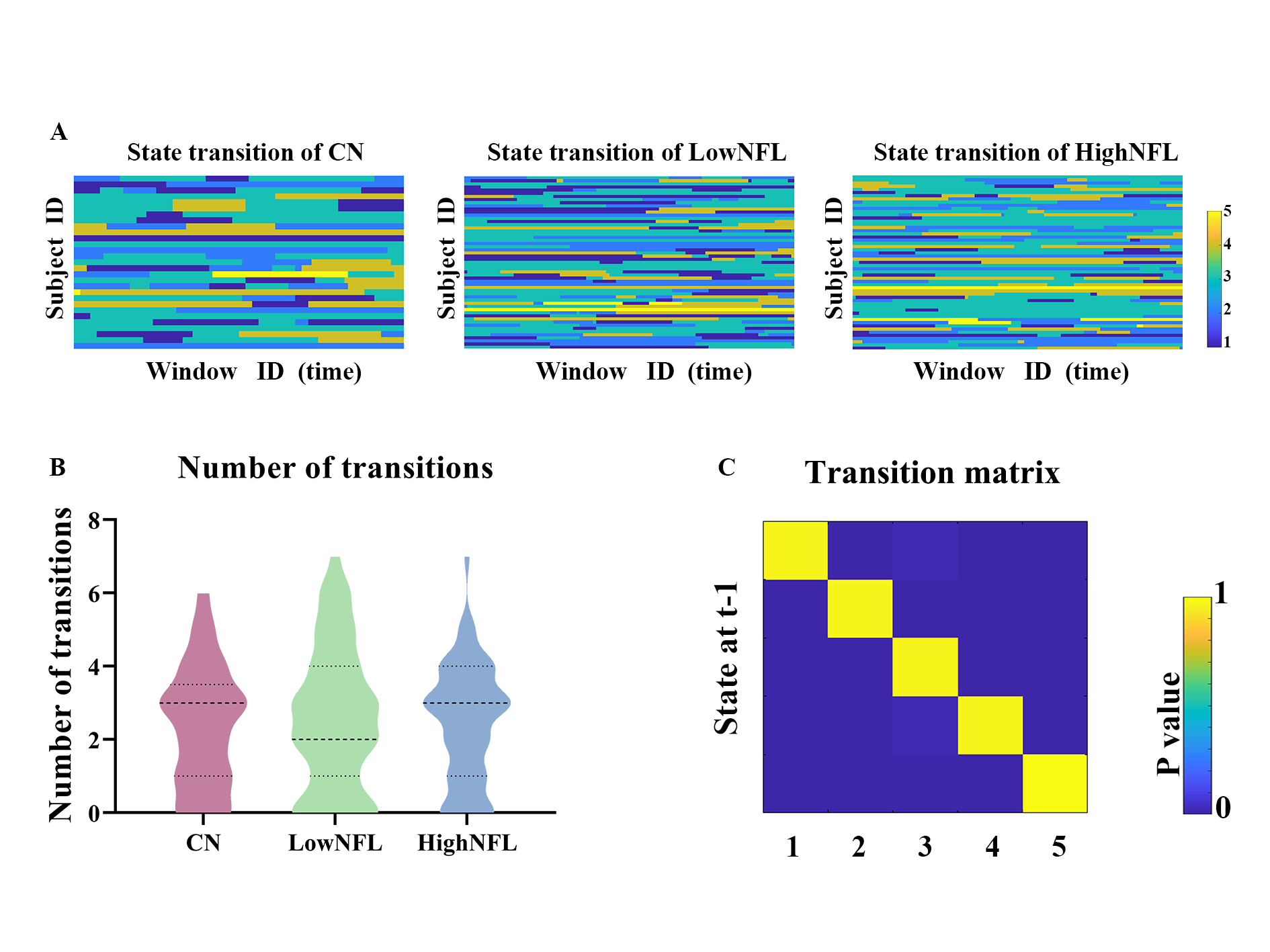


**Figure S2.** Between-group differences in the variances of network efficiency. The variances of (A) global efficiency and (B) local efficiency shown with violin plots. Asterisks represent significant differences at p < 0.05.


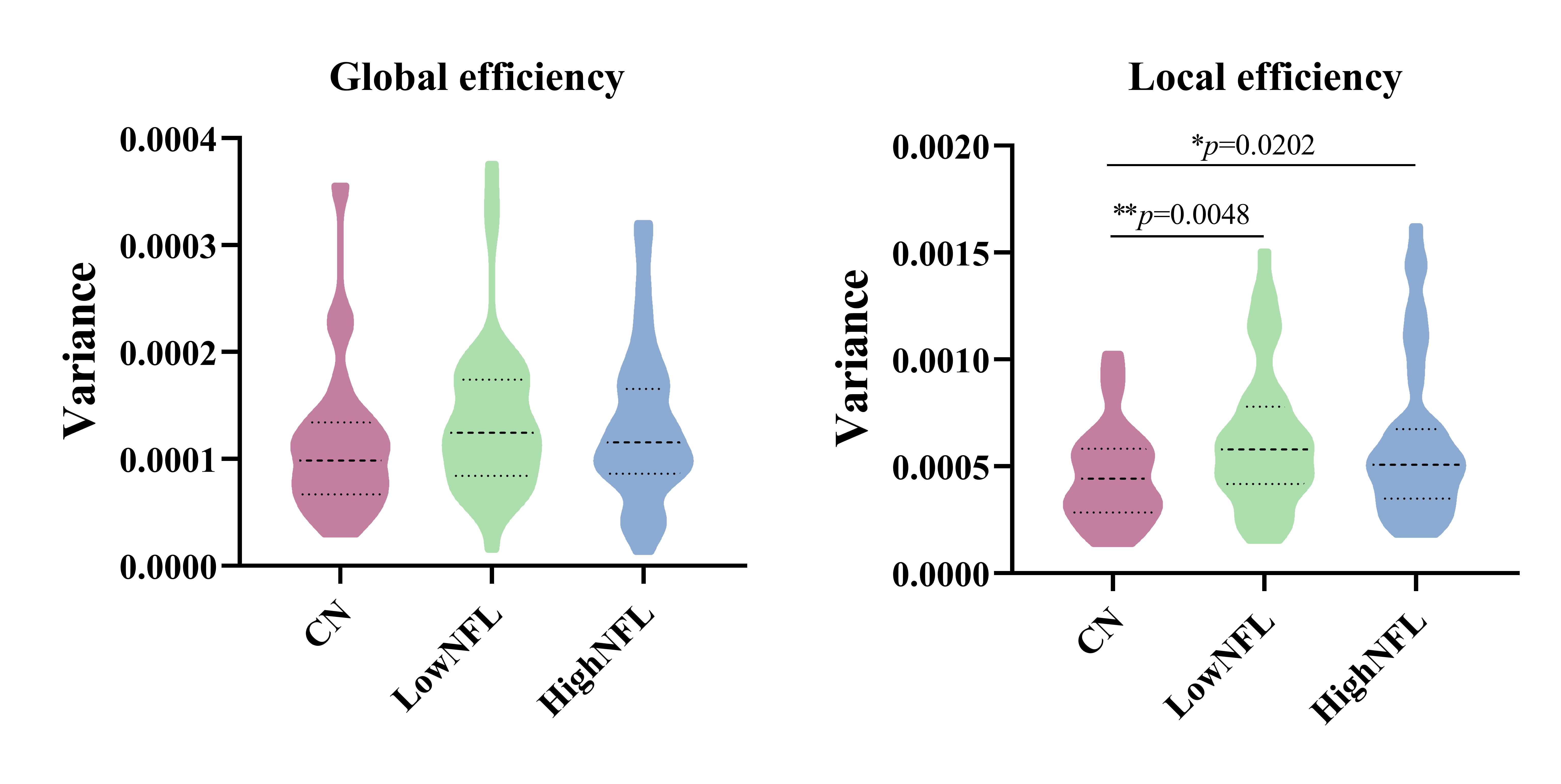

Supplement: Supplementary file 1 — Appendix S1 [file CNS-30-e14391-s001.doc]
